# Brain gliomas: reporting essentials and treatment response

**DOI:** 10.1186/1470-7330-15-S1-O39

**Published:** 2015-10-02

**Authors:** Bradley J Erickson

**Affiliations:** 1 Department or Radiology, Mayo Clinic, Rochester, MN, 55902, USA

## 

The diagnosis of brain glioma can challenging, particularly when the masses are very large. While the majority of gliomas are astrocytomas or oligodendrogliomas, enough are of other types that awareness of these others is critical. This course will begin with a review of some of the distinctive features of common and less common brain tumours, with particular focus on the distinctions that make a difference in approach.

Once a tumour is diagnosed and therapy instituted, clinicians will appreciate it greatly if you interpret the images with a mind to how they measure therapy response. This requires an awareness of the treatment regimen the patient is on, and changes in agents like steroid dose.

RECIST is applied throughout most of the body, but recently, the Response Assessment in NeuroOncology (RANO) criteria were published, to address some of the specifics of neurooncology [1]. These are still largely visually based, and some are retrospective, designed for clinical trials, not well-suited for patient management. If the patient is on trial, the referring physician may wish to have RANO-based assessments, but in cases where they are not on protocol, other styles of reporting are likely to be more valuable.

RANO is largely focused on conventional anatomic imaging methods, but new quantitative methods reflecting water diffusion and tumour perfusion appear to improve response assessment. Diffusion restriction typically reflects higher cell density seen in viable tumour as well as higher grade tumour [2]. Changes in apparent diffusion coefficient (sometimes referred to as functional diffusion mapping) can be very helpful in understanding an imaging examination [3]. Higher cerebral blood volume is also seen in viable tumour and higher grade tumours. When conventional, diffusion and perfusion all agree, the confidence in one's assessment can be high and some have proposed mathematical combinations to further improve diagnostic performance [4,5]. However, they often do not agree, and it is critical to be aware of the limitations and pitfalls in these methods that might lead to a contradiction and an error in assessment.

The combination of temozolomide and radiation has been shown to improve survival but also has a high rate of pseudoprogression, which is present in 1/3 to 1/2 of subjects, particularly when the tumour is MGMT methylated [6]. It is important to distinguish pseudoprogression from true progression so that patients can be maintained on effective therapy. Patients with true progression are often switched to anti-angiogenic agents that can dramatically reduce enhancement and cerebral blood volume, suggesting response, when the tumour is still growing (pseudoresponse). Imaging findings that can help to diagnose pseudoresponse will also be discussed. Newer agents like measles vaccine can also produce imaging findings that can be confusing and confounding, and examples will be presented.
